# The calcium-activated slow AHP: cutting through the Gordian knot

**DOI:** 10.3389/fncel.2012.00047

**Published:** 2012-10-25

**Authors:** Rodrigo Andrade, Robert C. Foehring, Anastasios V. Tzingounis

**Affiliations:** ^1^Department of Pharmacology, Wayne State University School of MedicineDetroit, MI, USA; ^2^Department of Anatomy and Neurobiology, The University of Tennessee Health Science CenterMemphis, TN, USA; ^3^Department of Physiology and Neurobiology, University of ConnecticutStorrs, CT, USA

**Keywords:** Ca^2+^-activated afterhyperpolarization, sAHP, KCNQ, PtdIns(4,5)P_2_, neuromodulation, pyramidal cell

## Abstract

The phenomenon known as the slow afterhyperpolarization (sAHP) was originally described more than 30 years ago in pyramidal cells as a slow, Ca^2+^-dependent afterpotential controlling spike frequency adaptation. Subsequent work showed that similar sAHPs were widely expressed in the brain and were mediated by a Ca^2+^-activated potassium current that was voltage-independent, insensitive to most potassium channel blockers, and strongly modulated by neurotransmitters. However, the molecular basis for this current has remained poorly understood. The sAHP was initially imagined to reflect the activation of a potassium channel directly gated by Ca^2+^ but recent studies have begun to question this idea. The sAHP is distinct from the Ca^2+^-dependent fast and medium AHPs in that it appears to sense cytoplasmic [Ca^2+^]_i_ and recent evidence implicates proteins of the neuronal calcium sensor (NCS) family as diffusible cytoplasmic Ca^2+^ sensors for the sAHP. Translocation of Ca^2+^-bound sensor to the plasma membrane would then be an intermediate step between Ca^2+^ and the sAHP channels. Parallel studies strongly suggest that the sAHP current is carried by different potassium channel types depending on the cell type. Finally, the sAHP current is dependent on membrane PtdIns(4,5)P_2_ and Ca^2+^ appears to gate this current by increasing PtdIns(4,5)P_2_ levels. Because membrane PtdIns(4,5)P_2_ is essential for the activity of many potassium channels, these finding have led us to hypothesize that the sAHP reflects a transient Ca^2+^-induced increase in the local availability of PtdIns(4,5)P_2_ which then activates a variety of potassium channels. If this view is correct, the sAHP current would not represent a unitary ionic current but the embodiment of a generalized potassium channel gating mechanism. This model can potentially explain the cardinal features of the sAHP, including its cellular heterogeneity, slow kinetics, dependence on cytoplasmic [Ca^2+^], high temperature-dependence, and modulation.

In many types of neurons, Ca^2+^-activated potassium currents mediate afterhyperpolarizing potentials (AHPs) that play important roles in shaping action potentials and firing patterns (Hille, 2001; Sah and Faber, 2002; Vogalis et al., 2003; Bean, 2007). Work during the last two decades has identified the ion channels carrying some of these currents and elucidated the mechanisms underlying their gating by Ca^2+^ as well as their modulation. One of these currents, however, the aptly named slow Ca^2+^-activated potassium current (I_sAHP_) has remained a conspicuous laggard in both of these regards. This current was originally identified in pyramidal cells of hippocampus and cortex and has been implicated in the control of repetitive firing including spike frequency adaptation (Sah and Faber, 2002; Vogalis et al., 2003), the setting of a neuron's dynamic firing range and the regulation of neuronal gain (Higgs et al., 2006). Yet, in spite of a well appreciated functional importance, the elucidation of its molecular basis has proven remarkably elusive. Most notably, in spite of considerable effort there remains considerable incertitude regarding how Ca^2+^ gates this current and about the molecular identity of the channels carrying it. In this article, we review past work on the slow afterhyperpolarization (sAHP) and its underlying current and highlight some of the difficulties encountered when trying to understand this current as resulting from the activation of a canonical calcium-activated potassium channel. We then focus on more recent studies that have begun to sketch a possible model capable of explaining the unusual properties of this enigmatic Ca^2+^-activated potassium current.

## Early studies

In the early 1980's, several studies reported that strong stimuli capable of triggering trains of action potentials elicited a long lasting AHP in many neurons including pyramidal cells of the CA1 and CA3 subfields of the hippocampus, neurons of the locus coeruleus, the nucleus of solitary tract, and myenteric neurons (Alger and Nicoll, 1980; Hotson and Prince, 1980; Schwartzkroin and Stafstrom, 1980; Gustafsson and Wigstrom, 1981; Madison and Nicoll, 1982; Morita et al., 1982; Brown and Griffith, 1983; Haas and Konnerth, 1983; Andrade and Aghajanian, 1984; Dekin and Getting, 1984; Williams et al., 1984; Pennefather et al., 1985; Lancaster and Adams, 1986; Storm, 1990). These AHPs could be shown to be Ca^2+^-dependent (Alger and Nicoll, 1980; Hotson and Prince, 1980; Schwartzkroin and Stafstrom, 1980; Morita et al., 1982; Andrade and Aghajanian, 1984; Hille, 2001) and to reflect the activation of a K^+^ selective current (Alger and Nicoll, 1980; Andrade and Aghajanian, 1984; Williams et al., 1984). Most distinctively, they all exhibited remarkably slow activation and decay that distinguished them from other AHPs known at the time from work in muscle cells and invertebrate neurons (Meech, 1978).

**Figure 1:**
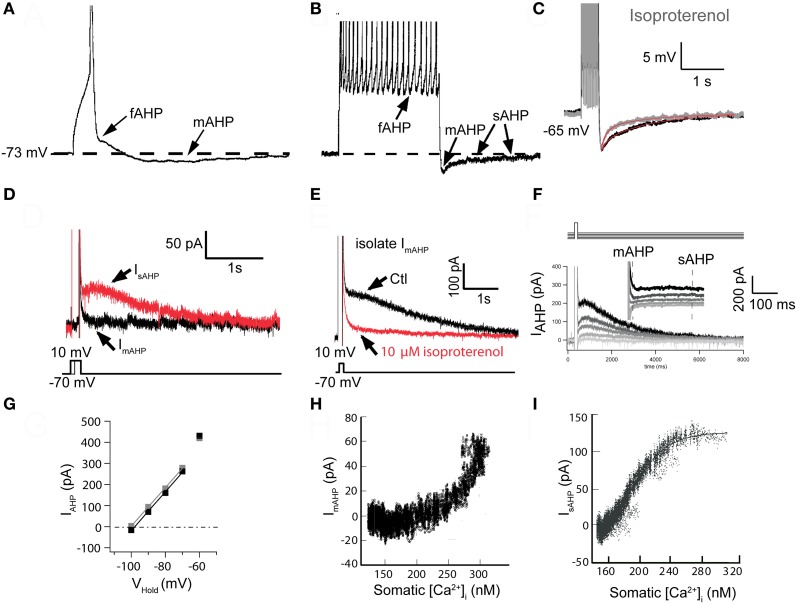
**The slow sAHP and underlying current (I_sAHP_) in neocortical pyramidal neurons from somatosensory cortex. (A)** The three AHPs. A single action potential (AP) was elicited with a 10 ms suprathreshold intracellular current injection (spikes truncated by digitization to emphasize afterpotentials). Note the notch following AP repolarization (the fast AHP: fAHP) and subsequent medium AHP (mAHP). **(B)** Data from the same cell as in **(A)**, except 10 APs were elicited with 10 ms suprathreshold current injections [@ 50 Hz, Panels **A** and **B** are redrawn from results presented in Pineda et al. (1998)]. Following the train of APs, two AHP components are evident: the mAHP is the main determinant of the initial peak response. A much slower decaying (τ > 1 s) slow AHP (sAHP) follows (spikes truncated by digitization to emphasize afterpotentials). **(C)** The sAHP elicited by 1 s repetitive firing is reduced in the presence of the β-agonist isoproterenol [10 μ M: modified from Figure 5A in Abel et al. (2004)]. **(D)** Tail currents were elicited following voltage steps from −70 mV to 0 mV for different durations. Following the 20 ms step (black trace), only I_fAHP_ and I_mAHP_ were observed upon return to −70 mV. The longer, 150 ms step (red trace) elicited both an initial I_mAHP_ and I_sAHP_. Note the slow time to peak (the peak occurs well after the voltage step) and slow decay of the sAHP [τ > 1 s: modified from Figure 7B in Abel et al. (2004)]. **(E)** In a different cell, the I_sAHP_ tail current following a 200 ms voltage step to 0 mV and then returning to −70 mV was blocked by 10 μ M isoproterenol, isolating I_mAHP_ [modified from Figure 7C in Abel et al. (2004)]. **(F)** Reversal potential for I_sAHP_. Tail currents were elicited by a 200 ms step to +10 mV and amplitudes were measured upon return to various potentials. I_mAHP_ was measured as the peak response and I_sAHP_ was measured at 500 ms after the peak, when I_mAHP_ had completely decayed [modified from Figure 8A in Abel et al. (2004)]. **(G)** Plots of I_AHP_ amplitudes from data in **(F)**. Extrapolated reversal potentials approximated *E*_*K*_, as determined by the Nernst equation [*E*_*K*_ = −102 mV: modified from Figure 8B in Abel et al. (2004)]. **(H)** Plot of isolated I_mAHP_ vs. bulk cytoplasmic [Ca^2+^]_i_. Since the underlying SK channels respond to a sub-membrane microdomain of [Ca^2+^], the dose-response relationship is distorted [data from eight cells; modified from Figure 10C in Abel et al. (2004)]. **(I)** Plot of isolated I_sAHP_ vs. bulk cytoplasmic [Ca^2+^]_i_. Note the sigmoidal dose-response curve indicating response to a “well-mixed” bulk [Ca^2+^]_i_ [data from five cells; estimated *K*_*D*_ = ~200 nM, Hill coefficient ~4.5: modified from Figure 9C in Abel et al. (2004)]. Panels **A** and **B** were from layer 5A of somatosensory cortex. Panels **C**–**I** were from layer 2/3.

In CA1 pyramidal cells, Storm (1987, 1989, 1990) described three AHP components following action potentials that were termed the fast, medium, and slow AHP, respectively. Three distinct AHP components were subsequently described in cat cerebral cortex using combined current- and voltage-clamp recordings (Schwindt et al., 1988a,b). In both pyramidal cell types the fast AHP (fAHP) was defined as the early component that followed the repolarization of an action potential (Figures 1A,B). This fAHP was followed by a more slowly decaying component that could also follow a single action potential and was named the medium AHP (mAHP: Figures 1A,B). Finally, there was a delayed component, the slow AHP (sAHP), which was evident only after a burst of spikes and could be distinguished by its strong regulation by neuromodulators (Figure 1C).

The existence of these three AHP components was later confirmed by multiple studies in rodent and human neocortex (Lorenzon and Foehring, 1992, 1993), and several other cell types (Viana et al., 1993; Pape and Driesang, 1998; Teruyama and Armstrong, 2005), although the relative expression of these components, and their corresponding currents were found to vary between cell types. These studies also revealed that the fAHP and the mAHP, as defined by their kinetics, consisted of Ca^2+^-dependent as well as calcium-independent components (Storm, 1987, 1989, 1990; Schwindt et al., 1988a,b; Pineda et al., 1992; Miles et al., 2005; Pedarzani and Stocker, 2008). The Ca^2+^-activated component of the fAHP was found to be mediated by large conductance BK-type channels (Lancaster and Nicoll, 1987; Storm, 1987, 1990; Sah and McLachlan, 1992; Miles et al., 2005; Ghatta et al., 2006) while the Ca^2+^-activated component of the mAHP, at least in neocortical pyramidal cells, was shown to be apamin sensitive indicating it is mediated by small-conductance calcium activated potassium channels (SK, now known as KCa2; Schwindt et al., 1988a,b; Lorenzon and Foehring, 1992; Pineda et al., 1992). In contrast, the sAHP appeared to be consistently Ca^2+^-dependent suggesting a unitary mechanism. Interestingly, in CA1 pyramidal neurons, the mAHP does not appear to have a Ca^2+^- or apamin-sensitive component (Storm, 1989; Gu et al., 2005, 2008), despite the presence of clear SK-mediated currents in response to voltage steps (Sah and Clements, 1999; Stocker et al., 1999). It should also be mentioned that the latter part of the sAHP in cat neocortical pyramidal neurons was not Ca^2+^-dependent but rather appeared due to a Na^+^-dependent potassium conductance (Foehring et al., 1989; Schwindt et al., 1989). The basis for this Na^+^-dependent conductance is beyond the scope of the present review.

In the absence of specific blockers for the sAHP, the strongest indication that this AHP component reflected the activation of a distinct calcium-activated potassium current came from the observation that the sAHP, unlike the fAHP or mAHP, was highly susceptible to neuromodulation. This was initially demonstrated for norepinephrine, acting through β-adrenergic receptors (Madison and Nicoll, 1982), and histamine acting via H_2_ receptors (Haas and Konnerth, 1983), both of which inhibited the sAHP and decreased spike frequency adaptation in pyramidal neurons of the CA1 region of the hippocampus. Subsequent studies extended these observations to other cell types and for other transmitters that activate Gα_s_-coupled receptors leading to increases in cAMP and activation of protein kinase A (PKA, Figures 1C,E; e.g., Andrade and Nicoll, 1987; McCormick and Prince, 1988; Foehring et al., 1989; McCormick and Williamson, 1989; Pedarzani and Storm, 1993, 1995; Torres et al., 1995; Pedarzani et al., 1998; Haug and Storm, 2000; Lancaster et al., 2006) or that activate Gα_q−11_ leading to the activation of phospholipase C and the breakdown of membrane phosphatidylinositol 4,5-biphosphate (PtdIns(4,5)P_2_, Dutar and Nicoll, 1988; Krause et al., 2002; Villalobos et al., 2011). In fact most known neuromodulators and neurotransmitters acting through receptors coupling to these canonical signaling cascades have been shown to inhibit the sAHP (Benardo and Prince, 1982; Cole and Nicoll, 1984; Lancaster and Nicoll, 1987; Madison et al., 1987; Schwindt et al., 1988a; McCormick and Williamson, 1989; Charpak et al., 1990; Araneda and Andrade, 1991; Lorenzon and Foehring, 1992; Torres et al., 1996; Villalobos et al., 2005).

## The search for the elusive sAHP channel

Early studies showed that the reversal potential for the sAHP/I_sAHP_ was dependent on extracellular potassium concentration in a manner predicted by the Nernst equation (Figures 1D–G; Alger and Nicoll, 1980; Andrade and Aghajanian, 1984; Williams et al., 1984; Lancaster and Adams, 1986; Constanti and Sim, 1987; Schwindt et al., 1988b; Lorenzon and Foehring, 1992). The sAHP was also clearly activated by an elevation in intracellular [Ca^2+^]. Thus, the sAHP was blocked by extracellular application of inorganic calcium channel blockers (e.g., Cd^2+^ or Co^2+^; Alger and Nicoll, 1980; Hotson and Prince, 1980; Morita et al., 1982; Andrade and Aghajanian, 1984; Madison and Nicoll, 1984; Schwindt et al., 1988b; Pineda et al., 1998) and by intracellular injection of Ca^2+^ chelators (Alger and Nicoll, 1980; Schwartzkroin and Stafstrom, 1980; Madison and Nicoll, 1984; Storm, 1987; Schwindt et al., 1988b; Lorenzon and Foehring, 1995; Velumian and Carlen, 1999). Similarly the sAHP was also activated by photolytic release of Ca^2+^ (Lancaster and Zucker, 1994; Sah and Clements, 1999) and inhibited by photolytic Ca^2+^ chelation (although not rapidly, Lancaster and Zucker, 1994; Sah and Clements, 1999). These observations indicated that the sAHP is mediated by the activation of a Ca^2+^-activated potassium current. However, these results provide only limited guidance as to the molecular identity of the channel carrying the I_sAHP_.

The search for the ion channel mediating the sAHP coincided with the molecular identification of ion channel families during the 1990s. At the time the expectation was that the channel responsible for I_sAHP_ would turn out to be a potassium channel directly gated by Ca^2+^ and the discovery of the *Drosophila* Ca^2+^-activated potassium channel *Slo* initially supported this idea (Adelman et al., 1992; Bond et al., 2004; Salkoff et al., 2006). The subsequent identification of SK channels seemed initially to identify a plausible candidate channel family capable of carrying I_sAHP_ (Bond et al., 2004). Specifically SK1 channels were initially reported to exhibit a lower sensitivity to apamin (Kohler et al., 1996), raising the possibility that such channels could mediate the sAHP. This observation led to the explicit proposal that SK1 channels, in association with delayed facilitation of L-type calcium channels, could be responsible for the sAHP in CA1 pyramidal neurons (Bowden et al., 2001). Subsequent work, however, questioned the apamin insensitivity of SK1, casting doubts on this possibility (Shah and Haylett, 2000b; Grunnet et al., 2001; Weatherall et al., 2010). Nevertheless, it remained possible that SK channels could be formed with unique stoichiometries or co-assemble with additional subunits to render them insensitive to apamin. To address these uncertainties two independent groups used dominant negative and overexpression approaches (Villalobos et al., 2004) or gene deletion strategies (Bond et al., 2004) to target SK channels in pyramidal neurons. Both studies confirmed the role of SK channels in carrying the current responsible for the mAHP but could find no evidence that these channels participated in the generation of I_sAHP_. Thus, I_sAHP_ was clearly not carried through BK or SK channels. So what are the molecular underpinnings of I_sAHP_?

## Clues from the activation of the sAHP by Ca^2+^

During neuronal firing intracellular Ca^2+^ increases with the number of spikes until a plateau is attained where there is a balance between Ca^2+^ entry and extrusion (Regehr et al., 1994; Helmchen et al., 1996; Maravall et al., 2000; Abel et al., 2004). Consequently the amplitude of the Ca^2+^-activated AHP is strongly dependent on neuronal activity and summates non-linearly as the action potential firing frequency increases. However, there are important differences in how Ca^2+^ activates the different components of the AHP.

Ca^2+^ entering a cell through calcium channels during one or more action potentials creates transient nanodomains of high calcium concentration that can sustain the activation of low affinity BK channels (μM EC_50_; Fakler and Adelman, 2008). As Ca^2+^ diffuses away from the calcium channel and the plasma membrane it combines with Ca^2+^ from other channels to create larger microdomains of elevated Ca^2+^ (Neher, 1998; Fakler and Adelman, 2008). Thus, Ca^2+^ flowing through multiple calcium channels are thought to contribute to the formation of these microdomains. Following termination of the Ca^2+^ influx, diffusion and the interaction with intracellular Ca^2+^ reaction partners leads to the dissipation of the [Ca^2+^] gradient associated with these microdomains and equilibration with the bulk cytoplasm. Bulk cytoplasmic [Ca^2+^] remains elevated until Ca^2+^ is taken up into intracellular stores or extruded from the plasma membrane. An important difference between the mAHP and the sAHP concerns how they are activated by the different Ca^2+^ pools resulting from these processes.

In expression systems, SK channels respond rapidly to elevations in intracellular [Ca^2+^] and there is a sigmoidal and cooperative relationship between [Ca^2+^] and the macroscopic SK current (Kohler et al., 1996; Hirschberg et al., 1998). Wilson and Callaway (2000) considered the relationship between the apamin sensitive (SK-mediated) I_AHP_ versus bulk intracellular [Ca^2+^] in dopaminergic neurons of the substantia nigra and concluded that a sigmoidal dose-response relationship between I_AHP_ and bulk cytoplasmic [Ca^2+^] would occur only if cytoplasmic [Ca^2+^] was well mixed. Since such a situation is unlikely to occur near the membrane immediately after Ca^2+^ entry, when [Ca^2+^] would be highest at the membrane and lower in the cytoplasm, or subsequently as pumps lower [Ca^2+^] near the plasma membrane, this provided an avenue for assessing the location of the activating Ca^2+^. They observed a distorted sigmoidal relationship between bulk [Ca^2+^] and the apamin sensitive AHP in dopamine cells, as did Abel et al. (2004) for I_mAHP_ in neocortical pyramidal cells (Figure 1H). These results suggest that apamin-sensitive mAHP responds to restricted microdomains of Ca^2+^ not accurately reflected by measurement of bulk [Ca^2+^]. This is consistent with the previous demonstration that SK channels sense Ca^2+^ through their constitutive association with calmodulin (Xia et al., 1998). In contrast, the sAHP current exhibits a cooperative and sigmoidal dose-response relationship with bulk cytoplasmic calcium (Figure 1I, Abel et al., 2004). In other words, the sAHP channels in neocortical pyramidal cells integrate a Ca^2+^ signal that is proportional to that measured in the bulk cytoplasm. These results suggest a cytoplasmic localization for the sAHP Ca^2+^ sensor.

If the sAHP activation reflects the rise in bulk calcium, it could be expected to exhibit a loose coupling to calcium channels. Consistent with this idea, the relationship of the sAHP to specific calcium channel subtypes is not strict and the coupling between specific calcium channels and the sAHP appears to be cell type-specific. For example, pyramidal cells of the cerebral cortex express a large sAHP during early postnatal development that is activated, at least in part, by L-type channels and ryanodine-sensitive internal stores (Lorenzon and Foehring, 1993; Pineda et al., 1999). However, in mature neocortical pyramidal neurons the sAHP is activated instead by N- and P/Q-type but not by L-type channels (Pineda et al., 1998). More generally, it is now clear that practically all calcium channel classes can activate the sAHP. Thus, for example, N-type calcium channels have been shown to couple to the sAHP in vagal motoneurons (Sah, 1995), superior cervical ganglion (Maingret et al., 2008), AH-type myenteric neurons from duodenum (Vogalis et al., 2001), and mouse sympathetic neurons (Martinez-Pinna et al., 2000b). Similarly L-type channels have been reported to contribute to the activation of the sAHP in CA1 (Moyer et al., 1992) and CA3 (Tanabe et al., 1998) pyramidal cells of the hippocampus, and in cholinergic interneurons of the striatum (Goldberg and Wilson, 2005; Gamelli et al., 2011). L- and N-type channels both contribute to the activation of the sAHP in CA1 pyramidal cells in culture (Shah and Haylett, 2000a) as well as dentate granule cells (Aradi and Holmes, 1999). Finally, L-, N-, P-type channels have been reported to activate the sAHP in guinea pig sympathetic neurons (Martinez-Pinna et al., 2000a), while T-type calcium channels can activate the sAHP in thalamic paraventricular nucleus (Zhang et al., 2009). Ryanodine-sensitive calcium stores have also been implicated in sAHP activation via calcium-induced calcium release in CA1 and CA3 neurons (Torres et al., 1996; Tanabe et al., 1998; Shah and Haylett, 2000a), guinea pig sympathetic neurons (Jobling et al., 1993) and vagal motoneurons (Sah and McLachlan, 1991). Thus, these results indicate considerable promiscuity in the coupling Ca^2+^ sources to the sAHP. This is consistent with the idea that I_sAHP_ senses bulk cytoplasmic [Ca^2+^] and therefore is relatively unselective with respect to the origin of the Ca^2+^.

## The time course of the sAHP

A central feature of I_sAHP_ is that it activates very slowly after a spike train (hundred of ms). Specifically the I_sAHP_ rises much slower than the cytoplasmic [Ca^2+^] (Sah and Clements, 1999; Abel et al., 2004; Gerlach et al., 2004; Goldberg et al., 2009) and continues to rise after the peak of the calcium transient (Lasser-Ross et al., 1997; Jahromi et al., 1999). It also decays very slowly, up to several seconds in some cells. Historically several different possibilities have been proposed to account for these unusually slow kinetics.

The simplest idea that could explain the slow onset kinetics of the sAHP is that it reflects the slow equilibration of free Ca^2+^ in the cytosol. This explanation, however, is unlikely since free Ca^2+^ declines rapidly (~99% in first ms) due to binding to its reaction partners (Markram et al., 1998; Goldberg et al., 2009). Furthermore, activation of the sAHP by either neuronal depolarization or rapid Ca^2+^ uncaging results in similar time courses (Sah and Clements, 1999) and, at least in pyramidal cells and striatal cholinergic interneurons, there is a mismatch between the time course of the decay of I_sAHP_ and bulk [Ca^2+^] concentration in the soma or dendrites (Lasser-Ross et al., 1997; Jahromi et al., 1999; Abel et al., 2004; Goldberg et al., 2009). Finally changes in Ca^2+^ buffering can have differential effects on the time course of the calcium transient and I_sAHP_ (Schwindt et al., 1992b; Lorenzon and Foehring, 1995; Lasser-Ross et al., 1997; Jahromi et al., 1999).

Alternatively, the time course of activation could reflect the diffusion of Ca^2+^ ions from their source to distally located sAHP channels (Lancaster et al., 1991; Lancaster and Zucker, 1994; Zhang et al., 1995; Jahromi et al., 1999). This explanation, however, also seems unlikely. If we assume that Ca^2+^ diffusion distance determines onset kinetics and use the activation kinetics of BK or SK as benchmarks, it is possible to estimate the expected distance between calcium source and sAHP channels. Because of their low (μM) affinity for Ca^2+^, BK channels must be located within 10–20 nm of the Ca^2+^ source to be activated (Muller et al., 2007; Fakler and Adelman, 2008). In contrast, SK channels have a higher Ca^2+^ affinity (200–500 nM: Kohler et al., 1996; Xia et al., 1998) and thus can be effectively activated 50–100 nm from the Ca^2+^ source (Fakler and Adelman, 2008). The channels underlying the sAHP have similar affinity for Ca^2+^ as SK channels (Abel et al., 2004), but activate an order of magnitude slower. This indicates that the sAHP Ca^2+^ sensor would need to be located prohibitively far (100 s of nms) from the site of calcium entry to account for the slow activation of the current. Also inconsistent with this idea are studies using vagal motoneurons (Sah and McLachlan, 1991) and pyramidal cells (Sah and Isaacson, 1995; Lee et al., 2005) that have shown that the activation of the sAHP exhibits a high temperature sensitivity. Specifically, the sAHP has a Q_10_ between 2 and 4, a range of values that is inconsistent with aqueous diffusion and is usually associated with enzymatic activity or slow channel gating events. This argues against diffusion of Ca^2+^ as being the rate-limiting step for sAHP activation.

A third possibility is that the slow activation of the sAHP could reflect delayed facilitation of L-type calcium channels (Cloues et al., 1997; Bowden et al., 2001). In particular, the slow kinetics of the sAHP in CA1 pyramidal neurons has been attributed to delayed facilitation of L-type channels of the α1D (CaV1.3) type (Bowden et al., 2001). However, given the limited and partial role of L type calcium channels as calcium sources for the sAHP, this mechanism again seems unlikely to provide a universal account for the slow time course of activation of this current.

Finally, the slow activation of the sAHP current has also been attributed to slow binding of Ca^2+^ to its sensor, slow intrinsic kinetics of the sAHP potassium channel (Lancaster et al., 1991; Sah and Clements, 1999) or the involvement Ca^2+^-induced Ca^2+^ release (CICR) from internal stores. None of these factors alone, however, appears capable of fully accounting for the time course of the sAHP. For example the time to onset of the sAHP current has been shown to be insensitive to [Ca^2+^] casting doubts on the idea that slow calcium binding to the sensor could represent the rate-limiting step for activation of I_sAHP_ [(Sah and Clements, 1999; Gerlach et al., 2004), but see below]. Similarly, estimates of the sAHP channel mean open time based upon noise analysis are much too short to fully account for the slow onset kinetics of the current (Sah and Isaacson, 1995). Finally, although CICR does contribute to the sAHP in some neurons (Sah and McLachlan, 1992; Davies et al., 1996; Torres et al., 1996; Shah and Haylett, 2000a; Vogalis et al., 2001), it contributes little to the sAHP in other cell types including mature, repetitively firing neocortical pyramidal neurons (Zhang et al., 1995; Pineda et al., 1998) again casting doubt on the generality of such a mechanism.

The possibilities outlined above all have assumed that the channels underlying the sAHP are gated by Ca^2+^ in a relatively direct manner. Therefore, the solution to the anomalous properties of the sAHP/I_sAHP_ had to reside in the properties of the Ca^2+^ signal or the sAHP channel itself. More recent studies have focused on the possibility of more complex intermediate steps between Ca^2+^ influx and the activation of I_sAHP_. A recent study combining experimental and modeling approaches in striatal cholinergic interneurons has suggested a key role for intracellular Ca^2+^ buffering mechanisms in generating the slow time course of AHP currents. Using a computational approach this study revealed that non-equilibrium dynamics of Ca^2+^ redistribution among cytoplasmic binding sites with different Ca^2+^ binding kinetics can give rise to multiple timescales within the same cytoplasmic volume (Goldberg et al., 2009). Key to this model is the assumption that the sAHP Ca^2+^ binding site does not have direct access to cytoplasmic Ca^2+^ with a time course determined only by Ca^2+^ entry. Rather, the presence of other Ca^2+^ reaction partners with faster binding kinetics can shape the time course of calcium available to bind the sAHP sensor (Markram et al., 1998). The kinetics of the various reaction partners, not the rate of Ca^2+^ entry to the cell, would then determine the delivery of Ca^2+^ to the sAHP site. The Goldberg et al. (2009) analysis raises the possibility that the temporal properties of the sAHP, including the delay in its onset and its slow decay, may be caused by the kinetics of the sAHP Ca^2+^ binding site/sensor, interacting with alternate binding sites in the cytoplasm. This explanation is consistent with the sAHP's sensitivity to fast and slow exogenous buffers and its insensitivity to brief Ca^2+^ transients.

Alternatively, recent findings using molecular approaches have rekindled interest in the possibility that calcium may activate the sAHP indirectly, through a signaling cascade involving one or more intermediate step (Hocherman et al., 1992; Schwindt et al., 1992a; Zhang et al., 1995; Sah and Faber, 2002; Abel et al., 2004; Tzingounis et al., 2007; Villalobos and Andrade, 2010; Villalobos et al., 2011). An attractive feature of interposing one or more molecular steps between Ca^2+^ binding and channel activation is that such a mechanism can accomodate most of the puzzling features of the sAHP current including its slow kinetics and temperature-dependence, the multiple action potential requirement, and the fact that rather than responding to micro- or nanodomains of calcium, sAHP activation requires an elevation of Ca^2+^ in the cytoplasm.

## Neuronal calcium sensor proteins and the sAHP

**Figure 2:**
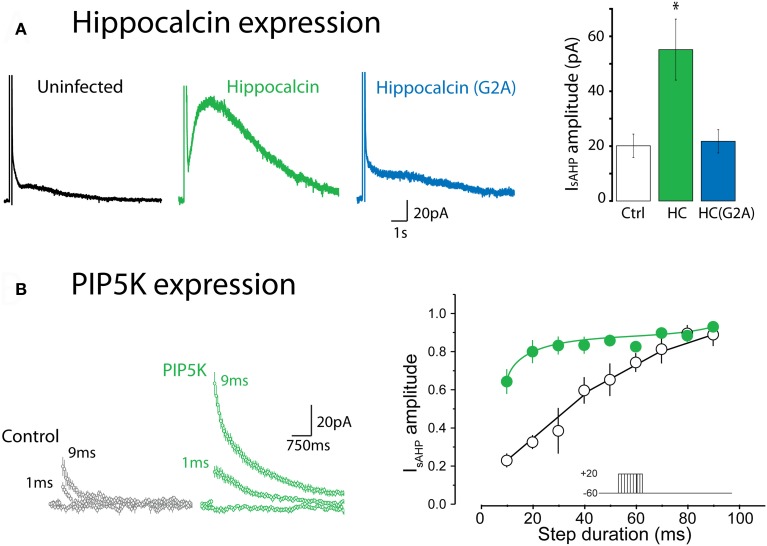
**Expression of hippocalcin and the phosphatidylinositol 4-phosphate 5-kinase (PIP5K) regulate I_sAHP_. (A)** Expression of wild type hippocalcin in hippocampal pyramidal cells in primary culture greatly enhances the amplitude of I_sAHP_. This enhancement is not seen with the G2A mutant, which cannot be myristoylated, thus pointing to an essential role for the translocation of hippocalcin to the plasma membrane. ^*^Indicates *p* < 0.001. Redrawn from data in Figure 4 of Tzingounis et al. (2007). **(B)** Expression of PIP5K enhances the apparent ability of calcium to elicit I_sAHP_. In this experiment calcium influx was titrated using depolarizing steps of increasing duration. Under control conditions I_sAHP_ is activated in a graded manner by depolarizing steps ranging from 10 to 100 ms. In contrast, in cells transfected with PIP5K I_sAHP_ is activated by much shorter steps. Redrawn from data in Figure 7 of Villalobos et al. (2011).

Recent experiments have provided strong evidence that hippocalcin, a member of the neuronal calcium sensor (NCS) protein family, is at least a partial Ca^2+^ sensor for the sAHP in pyramidal neurons (Tzingounis et al., 2007; Villalobos and Andrade, 2010). Hippocalcin is located in the cytoplasm but translocates to the plasma membrane upon Ca^2+^ binding with a relatively slow time course (Markova et al., 2008). This translocation is thought to result from a Ca^2+^-induced conformational change that leads to the exposure of a myristoyl group and repartition of the NCS to the plasma membrane. Tzingounis et al. (2007) found that I_sAHP_ was greatly reduced in a hippocalcin null mutant mouse and that expression of hippocalcin into cultured neurons enhanced the sAHP (Figure 2A). In these experiments hippocalcin appeared to act as a true Ca^2+^ sensor (as opposed to acting as a Ca^2+^ buffer) since I_sAHP_ was not enhanced by introduction of mutated hippocalcin with impaired myristoylation, and thus impaired translocation to the plasma membrane (Figure 2A). This last finding suggests that while hippocalcin may act as a mobile Ca^2+^ buffer relative to SK channels, its role in the sAHP is more as a true sensor. Collectively, these findings are consistent with the hypothesis that the Ca^2+^ sensor for the sAHP channels is not part of the channel complex but rather gates the sAHP upon translocation to the membrane. The need for such a mobile calcium sensor to translocate from the cytosol to the plasma membrane to activate the sAHP could help explain the slow time course of activation and dependence on bulk Ca^2+^.

In the Tzingounis et al. (2007) study, the sAHP was not completely eliminated in the hippocalcin knockout mouse. Furthermore, the distribution of hippocalcin in the brain only partly overlaps with the distribution of neurons exhibiting a pronounced sAHP [Allen Brain Atlas; (Villalobos and Andrade, 2010)]. A recent study indicated that in pyramidal cells of the prefrontal cortex, neurocalcin δ, a related neuronal Ca^2+^ sensor protein, acts similarly and perhaps in combination with hippocalcin to activate I_sAHP_ (Villalobos and Andrade, 2010). These results suggest that two or more NCS family members can act as calcium sensors linking rises in cytoplasmic Ca^2+^ to sAHP channel activation. A limitation of these studies is that they have relied upon constitutive gene deletions or overexpression strategies. This leaves open the possibility that the observed changes in the sAHP may reflect indirect effects of modifying the cells' ability to sense Ca^2+^.

## The elusive sAHP channel

A major impediment to identifying the channel carrying the sAHP has been the scarcity of pharmacological agents capable of selectively targeting this afterpotential. For instance, the sAHP in hippocampus and neocortical neurons is resistant to most known potassium channel blockers or toxins (e.g., apamin, TEA, 4-AP, cesium, quinine, ruthenium red). An important development was the discovery that clotrimazole (Shah et al., 2001) and especially its analog UCL2027 appear to be reasonably selective inhibitors of the sAHP, at least in some cell types (Shah et al., 2006; Lee et al., 2010). Besides the sAHP, the only known targets of UCL2027 are KCNQ-mediated currents (Soh and Tzingounis, 2010) suggesting the possible involvement of these channels in mediating the sAHP (see below). However, a more detailed investigation of the potassium channel selectivity of UCL2077 will be needed before UCL2077 can act as a screening tool for the sAHP channels.

In the absence of strong pharmacological leads several studies have used non-stationary noise analysis and single channel recordings to gain insight into the properties of the ion channels mediating the sAHP. Noise analysis in pyramidal cells of the CA1 region of the hippocampus (Sah and Isaacson, 1995), granule cells of the dentate gyrus (Valiante et al., 1997), and the dorsal motor nucleus of the vagus (Sah, 1995) revealed that potassium channels exhibiting a small conductance mediate the sAHP but there was considerable variability in their estimates for single channel conductance (γ: 2–10 pS). Potassium channels with γ in this range include SK (KCNN), some Kv1 (KCNA), Kv4 (KCND), some Kir (KCNJ) channels, and Kv7 (KCNQ) channels (Coetzee et al., 1999). It has recently been reported that in granule cells of the dentate gyrus, K_ATP_ channels open in response to action potential bursts and the resulting sAHP is reduced by the K_ATP_ inhibitor glibenclamide (Tanner et al., 2011). This suggests that these channels may contribute to the sAHP in granule cells. However, since γ is typically > >10 pS for K_ATP_ channels (Coetzee et al., 1999) such a mechanism is unlikely to be widely generalizable. Thus, collectively, these pharmacological and single channel studies fail to converge on a defined set of properties for the channels mediating the sAHP. The results suggest that I_sAHP_ may not be a unitary current due to a single molecular entity but rather may be mediated by a variety of ion channels depending on the cellular background.

An alternative approach to identify the channels responsible for the sAHP has been to examine the effect of ion channel subunit gene deletions on the sAHP. The KCNQ1–5 genes code for the Kv7 potassium channels that underlie the “M current” in a variety of central and peripheral neurons (Delmas and Brown, 2005). Surprisingly, the genetic deletion of KCNQ2 or KCNQ3 was found to result in a significant decrease in the amplitude of the sAHP current in granule cells of the dentate gyrus. Similarly, expression of a KCNQ2/3 pore-dead dominant negative in slice culture or of a KCNQ5 pore-dead dominant negative in a knock-in mouse both inhibited I_sAHP_ in CA3 pyramidal neurons (Tzingounis and Nicoll, 2008; Tzingounis et al., 2010). These results, along with the inhibition of the sAHP by UCL2077 (Soh and Tzingounis, 2010), suggest a significant role for KCNQ channels in the generation of the sAHP, at least in CA3 pyramidal and dentate granule cells.

The involvement of KCNQ channels in the generation of the sAHP has been controversial, at least in part, because KCNQ channels are inhibited, rather than activated by intracellular Ca^2+^. Previous studies, however, have shown that the calcium inhibition of KCNQ channels is mediated a Ca^2+^/calmodulin (CaM)-dependent mechanism (Selyanko and Brown, 1996; Gamper and Shapiro, 2003, reviewed by Delmas and Brown, 2005) while the activation of I_sAHP_ is mediated by NCS proteins of the hippocalcin family, which have much lower Ca^2+^ operating ranges than calmodulin [reviewed by Burgoyne (2007)]. The I_sAHP_ is activated by calcium with an EC_50_ ~ 300 nM, well within the operating range of hippocalcin but below that of calmodulin (O'Callaghan et al., 2003; Burgoyne, 2007). Therefore, the reported Ca^2+^-CaM inhibition of KCNQ channels is unlikely to operate during the I_sAHP_ as the Ca^2+^ levels necessary to activate the I_sAHP_ are well below those required for calmodulin to inhibit KCNQ channels. The idea that KCNQ channels may contribute to I_sAHP_ has also been questioned because the I_sAHP_ appears to be largely insensitive to KCNQ channel blockers in some of the prototypical cell types expressing this current. For example, in the CA1 region of the hippocampus, administration of KCNQ blockers (e.g., linopirdine or XE-991) has led to inconsistent results with some studies reporting partial block of I_sAHP_ (Schnee and Brown, 1998; Tzingounis and Nicoll, 2008), while others found no effect of these blockers to this current (Aiken et al., 1995; Gerlach et al., 2004; Gu et al., 2005). Similar studies on pyramidal cells from neocortex have also failed to find any effect of KCNQ channel blockers on I_sAHP_ (Abel et al., 2004; Guan et al., 2011). However, these observations are consistent with the possibility outlined above that I_sAHP_ may be mediated by different complement of channels in different cell types.

## PtdIns(4,5)P_2_ and the Ca^2+^-dependent regulation of potassium channels

Most of the channels considered above, including KCNQ, are not Ca^2+^-activated and some are also voltage activated. Therefore, if such channels were to underlie the sAHP a mechanism must exist to allow Ca^2+^ to indirectly activate them and/or alter their apparent voltage sensitivity. Recent work indicates that the functional expression of the sAHP current is dependent on membrane PtdIns(4,5)P_2_ and that increasing membrane PtdIns(4,5)P_2_ greatly facilitates the ability of Ca^2+^ to activate the sAHP (Figure 2B). This has been interpreted to suggest that Ca^2+^ acts upstream from PtdIns(4,5)P_2_ to activate I_sAHP_ (Villalobos et al., 2011). Since results from a variety of model systems indicate that Ca^2+^ can regulate the local availability of PtdIns(4,5)P_2_ in the membrane, a simple interpretation of these results is that Ca^2+^ gates the sAHP channels by increasing the availability of PtdIns(4,5)P_2_ near the channel. Previous studies have shown that PtdIns(4,5)P_2_ can control potassium channels at multiple levels. For example this phosphoinositide is required for the activity of many potassium channels of the K_ir_ and K_v_ families (Delmas and Brown, 2005; Hansen et al., 2011), can regulate the inactivation of “A type” potassium channels (Oliver et al., 2004) and modulate the affinity of K_ATP_ channels for ATP (Baukrowitz et al., 1998; Shyng and Nichols, 1998). Since many PtdIns(4,5)P_2_-sensitive potassium channels are subsaturated at rest, a transient Ca^2+^-triggered increase in PtdIns(4,5)P_2_ could be expected to result in an increase in the activity of multiple classes of potassium channels. At the macroscopic level this increase in channel activity would result in a slow potassium aftercurrent that could correspond to I_sAHP_ (Villalobos et al., 2011).

One of the attractive features of this mechanism is that it can explain with economy some of the most puzzling aspects of I_sAHP_. For example, the monoexponential decay of the sAHP (Lancaster and Adams, 1986) has been generally interpreted to indicate the involvement of a single type of channel in the generation of sAHP/I_sAHP_, even as growing evidence suggest considerable molecular diversity depending on the cell type examined (see above). A monoexponential decay implies a single mechanism functioning as the rate limiting step, which could reflect the involvement of a single ion channel subtype or a single (essential) biochemical step. Thus, a monoexponential decay that is independent of amplitude is equally well predicted by a model where activation of multiple types of potassium channels follows a rate-limiting intermediate step, in the current hypothesis the availability of PtdIns(4,5)P_2_. While these considerations suggest a broad range of potassium channels could participate in the generation of I_sAHP_, we believe that there still must be some molecular specificity as the sAHP channels have small single channel conductance and lack sensitivity to multiple known potassium channel blockers and toxins, features that are not shared by most potassium channels.

PtdIns(4,5)P_2_ by virtue of its effect on potassium channel gating may also help explain the involvement of channels that, based upon their voltage dependence, may appear unlikely candidates to carry I_sAHP_ (which is voltage-insensitive). Previous studies have shown that PtdIns(4,5)P_2_ stabilizes the open conformation of potassium channels including KCNQ channels (Enkvetchakul et al., 2000; Loussouarn et al., 2003; Park et al., 2005; Hernandez et al., 2009; Falkenburger et al., 2010; Rodriguez et al., 2010). Current models also suggest that KCNQ channels are gated allosterically by voltage, in other words that voltage sensor activation is not obligatory for channel opening. Consequently, a transient PtdIns(4,5)P_2_ increase might promote KCNQ channel opening at hyperpolarized potentials bypassing the need for multiple voltage sensor activation. A facilitation of such voltage independent transitions by PtdIns(4,5)P_2_ would manifest itself as a shift of the KCNQ channel half-activation voltage (V_0.5_) to more hyperpolarized values, leading to an apparent voltage insensitivity at the voltages where the sAHP is measured. Consistent with this possibility, recent work from Suh and Hille (2007) has shown that overexpression of the phosphatidylinositol 4-phosphate 5-kinase (PIP5K) in heterologous cells, which can be expected to increase basal PtdIns(4,5)P_2_ levels, shifts the KCNQ2/3 V_0.5_ to more hyperpolarized membrane potentials. Although this model is only a hypothesis it might provide a starting point for understanding the mechanism by which KCNQ channel or other voltage-activated potassium channels can contribute to the sAHP. A similar argument for the modulation of K_ATP_ channels by PtdIns(4,5)P_2_ can be based on a previously described model by Enkvetchakul et al. (2000). More broadly, this brief discussion highlights how the ability of PtdIns(4,5)P_2_ to regulate potassium channel gating could help explain some the properties of I_sAHP_.

Finally the PtdIns(4,5)P_2_ hypothesis also has the potential to help clarify the mechanisms underlying the modulation of I_sAHP_. The inhibition of a molecularly heterogeneous I_sAHP_ by receptors coupling to Gα_q−11_/PLCβ would simply follow from the lowering of membrane PtdIns(4,5)P_2_ levels (Villalobos et al., 2011). The inhibition of I_sAHP_ by activation of the Gα_s_/adenylate cyclase/cAMP/PKA signaling cascade is thought to involve a poorly understood phosphorylation step downstream from PKA (Pedarzani and Storm, 1993). Since PKA phosphorylation strongly inhibits PIP5K (Park et al., 2001), the rate limiting enzyme for the formation of PtdIns(4,5)P_2_, it seems possible that PKA may also inhibit I_sAHP_ by reducing membrane PtdIns(4,5)P_2_ levels. If this conjecture is correct it could explain how PKA activation could inhibit a current carried by ion channels, such as KCNQ, that are not directly modulated by cAMP/PKA.

**Figure 3:**
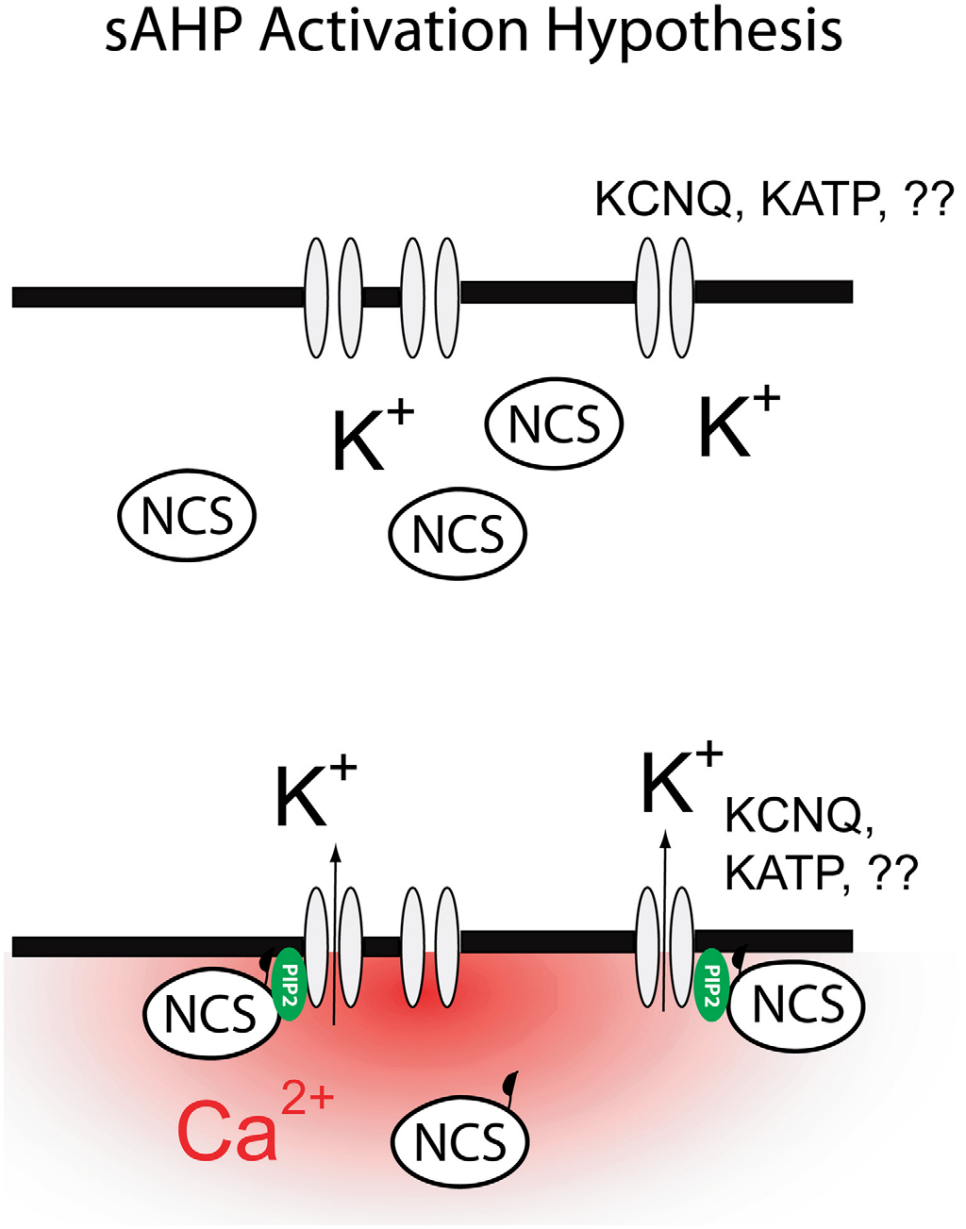
**Mechanism for the activation of the sAHP as proposed in this review.** Global increases in cytosolic calcium lead to activation of diffusible neuronal calcium sensors (NCS: hippocalcin, neurocalcin δ). Binding of calcium to NCS exposes a previously sequestered myristoyl moiety allowing NCS to bind to the plasma membrane. Binding of NCS to plasma membrane leads to a transient increase in PtdIns(4,5)P_2_ levels and subsequent activation of the sAHP.

The ideas outlined above are summarized in Figure 3. While this model offers a way forward in our thinking about the molecular physiology of the sAHP it is important to note that numerous important questions still remain even if this model proves correct. For example, which potassium channels are more likely to mediate the sAHP? Does the modulation of the PtdIns(4,5)P_2_ generating enzymes by kinases and phosphatases hold the answer to the neuromodulation of the sAHP by cAMP and PKA? How do NCS proteins gate the sAHP, do they bind directly to the sAHP channels or do they shuttle PtdIns(4,5)P_2_ generating enzymes to the plasma membranes? Fortunately we now have the molecular and conceptual tools for addressing these issues and thus it seems reasonable to expect quick progress on these and other questions central to our understanding the sAHP.

In summary, recent studies have begun to sketch a possible mechanism for I_sAHP_ involving the idea that Ca^2+^ gates I_sAHP_ indirectly, via a diffusible Ca^2+^ sensor and PtdIns(4,5)P_2_. While this idea still remains conjectural at this time, this conceptualization offers an economical way to reconcile some of the most puzzling effects of I_sAHP_ including its anomalous dependence on Ca^2+^, its slow kinetics and its apparent molecular heterogeneity depending on the cellular background. If these ideas are correct, perhaps after 30 years we may finally be cutting though the knot concealing the sAHP.

### Conflict of interest statement

The authors declare that the research was conducted in the absence of any commercial or financial relationships that could be construed as a potential conflict of interest.
